# Preliminary study on optimization of pH, oxidant and catalyst dose for high COD content: solar parabolic trough collector

**DOI:** 10.1186/1735-2746-10-13

**Published:** 2013-01-22

**Authors:** Chandan Singh, Rubina Chaudhary, Kavita Gandhi

**Affiliations:** 1 School of Energy and Environmental Studies, Devi Ahilya University, Indore, Takshashila Campus, Khandwa Road, Indore, M.P, India; 2 National Environmental Engineering Research Institute, Nehru Marg, Nagpur, India

**Keywords:** Synthetic high organic wastewater, Solar photocatalysis, Parabolic trough collector, Hydrogen peroxide

## Abstract

In the present study, solar photocatalytic oxidation has been investigated through laboratory experiments as an alternative to conventional secondary treatment for the organic content reduction of high COD wastewater. Experiments have been performed on synthetic high COD wastewater for solar photocatalytic oxidation using a parabolic trough reactor. Parameters affecting the oxidation of organics have been investigated.

The experimental design followed the sequence of dark adsorption studies of organics, followed by photolytic studies (in absence of catalyst) and finally photocatalytic studies in presence and absence of additional oxidant (H_2_O_2_). All the experimental studies have been performed at pH values of 2, 4, 6,8,10 and the initial pH value of the wastewater (normal pH). For photocatalytic studies, TiO_2_ has been used as a photocatalyst. Optimization of catalyst dose, pH and H_2_O_2_ concentration has been done. Maximum reduction of organic content was observed at the normal pH value of the wastewater (pH = 6.8). The reaction rate was significantly enhanced in presence of hydrogen peroxide. The optimum pH other than the Normal was in the alkaline range. Acidic pH was not found to be favourable for organic content reduction. pH was found to be a dominant factor affecting reaction rate even in presence of H_2_O_2_ as an additional oxidant. Also, the solar detoxification process was effective in treating a waste with a COD level of more than 7500 mg/L, which is a otherwise a difficult waste to treat. It can therefore be used as a treatment step in the high organic wastewater treatment during the primary stage also as it effectively reduces the COD content by 86%.

## Introduction

Industrial wastes, sewage and a wide array of synthetic chemicals, pollute considerable parts of water resources [1]. The elimination of toxic chemicals from wastewater is presently one of the most important aspects of pollution control. It causes problems to the classical biological treatment [2]. A wide range of organic compounds are detected in high organic wastewater. Some of these compound both synthetic organic chemical as well as naturally occurring substances, also pose severe problem in biological treatment [1].

The photocatalytic degradation method is fast, effective, ecofriendly, economically viable and efficient method in the waste water treatment [3]. Semiconductor photocatalytic process has shown a great potential as a low-cost, environmental friendly and sustainable treatment technology to align with the “zero” waste scheme in the water/wastewater [4]. The photocatalysis is one of the techniques, which are called "advanced oxidation processes (AOPs)". These processes can completely degrade the organic pollutants into harmless inorganic substances such as CO_2_ and H_2_O under moderate conditions [5].

It has been found that solar detoxification is one of the promising methods for the disinfection of the wastewater. Solar detoxification process uses sunlight as the primary energy input required in reactions that break down contaminant molecules in CO_2_ and water. The combination of light and catalysts has proven very effective for wastewater purification. The solar photocatalytic detoxification process uses the near ultraviolet band of the solar spectrum (wavelength under 390 nm) to promote oxidative / reductive reactions [6]. The solar photocatalytic wastewater treatment is based on reactive free potent chemical oxidants such as the hydroxyl radical.

The aim of the paper is to develop a method which takes care of energy and water conservation together. Solar photocatalytic oxidation of synthetic high organic wastewater is investigated in a trough collector with a parabolic reflector. Wastewater with a high COD of the order of more than 7500 mg/L is treated. The effect of parameters like optimum catalyst doses, pH and concentration of an additional oxidant (H_2_O_2_) has also been investigated, and to investigate the role of dark absorption of synthetic high organic wastewater constituents. The study will lead to a possibility of implementation of solar detoxification process as a treatment step for high organic wastewater.

## Materials and methods

### Study area

All the photoreaction experiments were performed under ambient conditions on the open roof of the building of School of Energy and Environmental Studies, Devi Ahilya University campus, located in Indore. The study area Indore (India) is situated at 22°43^′^ N, 75°48^′^ E. In most parts of India, clear sunny weather is experienced 250 to 300 days a year this makes India a suitable site for the solar-based treatment processes. The solar radiation intensity was 650 W/m^2^ during maximum experimental runs.

Average mean peak irradiance of Solar UV- A is 47 W/m^2^ to 66 W/m^2^ and average mean peak irradiance of Solar UV-B is 0.195 W/m^2^ to 0.3384 W/m^2^, corresponding to Indore field conditions [7].

### Experimental set-up

**Figure 1:**
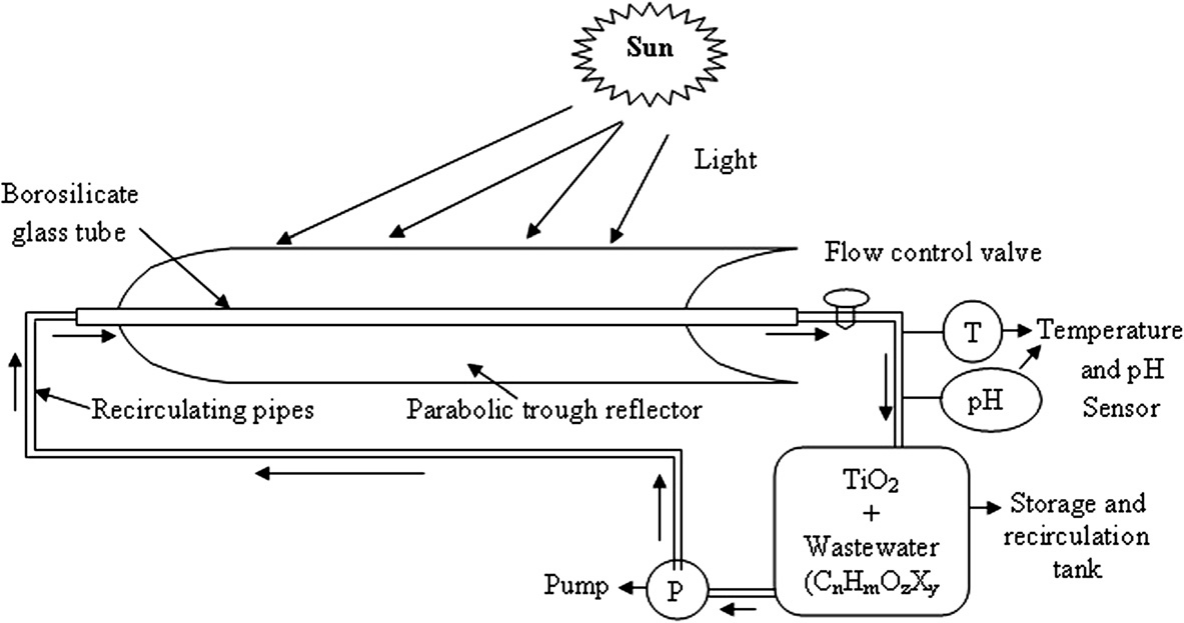
Schematic diagram of solar photocatalytic pilot plant reactors system (one parabolic trough reflector + tank + pump + connections).

All the experiments were carried out in a concentrating solar collector with a parabolic trough reflector. The photoreactor used was a transparent borosilicate glass tube with 38 mm internal diameter, 1.8 m length, mounted on a parabolic trough reflector of aperture length 172 cm and aperture width 57.75 cm. The system is shown in Figure 1. It maintains the turbulent conditions and there is no mass transfer limitation. It is a nearly closed system no vaporization of volatile compounds takes place. The size of the parabolic trough concentrator used in this study is even smaller when compared to the systems used by [8,9]. The temperature rise is also not very high (maximum 55–60°C) curtailing the need for any external cooling device.

The initial simulated volume of wastewater was 5 L for photocatalytic experiments. The reactor volume was 1 L. TiO_2_ was added in the form of a suspension, after collection of an initial sample of the wastewater. Thereafter, the samples were collected at regular intervals of 60 minutes. The time period of experiments was upto 300 minutes.

### Analytical procedure

The composition and preparation of synthetic high organic wastewater used for the study is given in Table 1[10] and its physicochemical characteristics are given in Table 2.

**Table 1:** Concentrated simulated influent composition of the organic wastewater

**Chemical compounds**	**mg/L**	**Food ingredients**	**mg/L**	**Trace metals**	**mg/L**
Urea	1581	Starch	2102	Cr(NO_3_)3.9H_2_O	13.27
NH_4_Cl	220	Milk powder	2002	CuCl_2_.2H_2_O	9.24
Na-acetate	1368	Yeast	900	MnSO_4_.H_2_O	1.86
Na-acetate.3H_2_O	2268	Soy oil	500	NiSO_4_.6H_2_O	5.79
Peptone	300			PbCl_2_	1.72
MgHPO_4_.3H_2_O	500				
KH_2_PO_4_	403				
FeSO_4_.7H_2_O	100				
ZnCl_2_	3.58				

**Table 2:** Simulated influent concentration

**Parameter**	**Concentration**
pH	6.5–7.5
mV	3–25
Conductivity (mS)	2.5–4
TDS (g/L)	150–250
Temperature (°C)	28–50
Light (W/m^2^)	625–850
COD (mg/L)	5500–7585

Chemical oxygen demand (COD) was measured according to the Standard Methods (APHA, 1995) under section 5220. TiO_2_ (Marck) photocatalyst was used as received. Anatase was the major crystalline phase in TiO_2_ as determined by XRD analysis (performed by rigaku RUH 3R instrument) and the surface area is 10.5 m^2^/g [11]. Solar intensity, temperature and pH were monitored regularly throughout the experiment. Solar intensity was measured using a solarimeter (Make- SM 201 Solar, Central Electronic Ltd., India). H_2_SO_4_ and NaOH were used to adjust the solution pH. Temperature and pH levels were monitored by using a digital temperature indicator and pH meter. COD was estimated before and after treatment.

## Results and discussion

### Photolytic reaction at different pH values

#### Effect of pH on dark absorption of COD on photolytic

**Figure 2:**
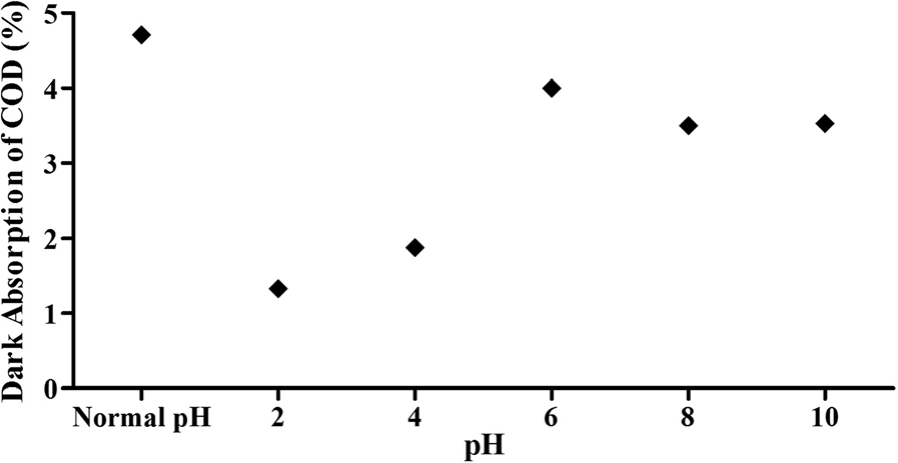
**Dark adsorption of organics on photocatalyst at different pH values. **Plot of COD concentration versus pH; initial COD concentration= >7500 mg/L; average temperature = 23°C

The effect of pH on dark adsorption of COD is shown in Figure 2. The sample used was similar to that of photoreduction experiments. It is clear that adsorption is maximum at Normal pH and consequently the degradation is also maximum at this pH. Adsorption was favoured more in the alkaline and neutral pH range [12].

#### Effect of pH on degradation of COD using solar irradiation

**Figure 3:**
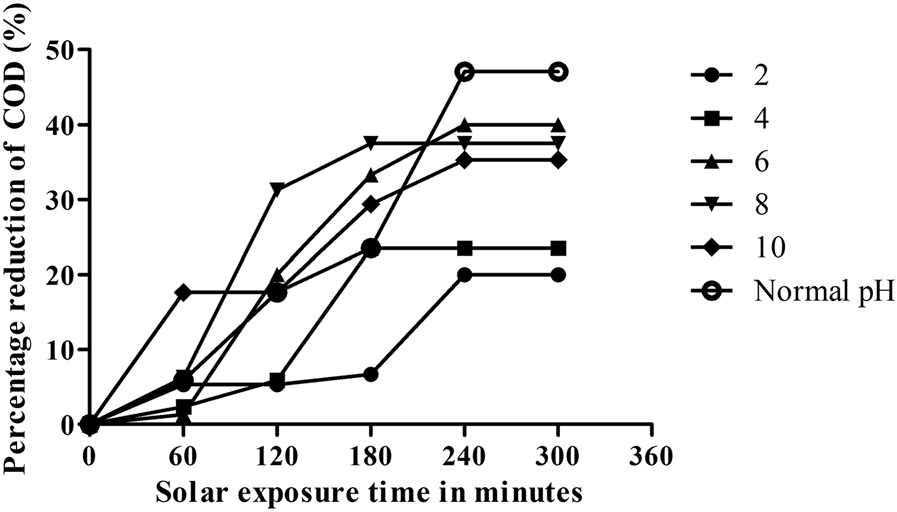
**Effect of pH on percentage reduction of COD using solar radiation. **Plot of COD reduction versus pH and time, initial concentration= >7500 mg/L; average solar intensity = 80 W/m^2^; average temperature = 30°C

Experiments were performed in absence of TiO_2_ to explore the possibility of photolytic reactions. Initially, the photolytic reactions were performed at the actual pH value of the wastewater without any pH alteration (pH = 6.8). This experiment was followed by the reaction at different pH values of 2, 4, 6, 8 and10. The results are depicted in Figure 3.

Maximum COD decrease of 47% was observed at Normal pH in 240 minutes. No further decrease was observed on performing the reaction till 300 minutes. As compared to acidic pH values, the decrease was more in the neutral and alkaline pH range. In 240 minutes, 40%, 37.5% and 35% decrease was observed at pH values 6, 8 and 10 respectively. The maximum degradation rate was obtained by simultaneous application of both the methods at neutral pH values [13]. The decrease in adsorption values may be due to the abundance of OH^-^ ions, causing increased hindrance to diffusion of organics [14].

The reaction rate was very slow in the acidic pH range. At pH = 4, 23.5% reduction in 180 minutes was observed. No further decrease was observed on carrying out the reaction upto 300 minutes. A 20% decrease in the initial COD value was observed at pH = 2 in 240 minutes.

### Effect of TiO_2_ concentration

#### Effect of TiO_2_ concentration on dark absorption of organics

**Figure 4:**
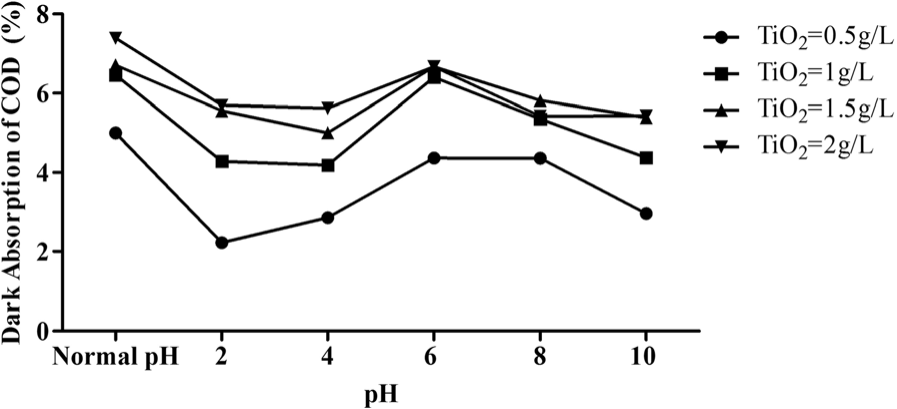
**Dark adsorption of organics at different pH values initial COD concentration = >7500 mg/L; TiO**_**2**_ **= variable; average temperature = 23°C.**

The catalyst concentration was varied as 0.5, 1, 1.5 and 2 g/L to obtain an optimum concentration to be used for further experiments. pH value was also altered for all the catalyst doses. Experiments were performed at Normal pH, 2, 4, 6, 8 and 10 values for all the mentioned catalyst concentrations. The effect of pH on dark adsorption of organic compounds is shown in Figure 4. The wastewater and catalyst concentration used was similar to that of photoreduction experiments. It is clear that adsorption is maximum at Normal pH in different TiO_2_ concentration (0.5, 1, 1.5 and 2 g/L) and consequently the degradation is also maximum at this pH as per the mechanism in which the adsorbed substrate reacts with ^·^OH on the TiO_2_ surface.

The effluent pH affects the surface of titania by protonation or deprotonation according to equations (1) and (2) [15].

(1) $${\mathrm{TiOH}}^{+}{\text{H}}^{+}\to {\mathrm{TiOH}}^{2}{}^{+}$$

(2) $${\mathrm{TiOH}}^{+}{\mathrm{OH}}^{-}\to {\mathrm{TiO}}^{-}+{\text{H}}_{2}\text{O}$$

A similar effect of effluent pH on the surface of TiO_2_, and they proposed the formation of three different species to account for variations of the behaviour of the catalyst with pH [16]. The species, namely TiOH, TiOH^2+^ and TiO^−^ (equations (1) and (4)) are formed on the amphoteric surface due to acid–base equilibria depending on the solution pH and the point-of-zero charge (pzc) of the catalyst.

(3) $${\mathrm{TiOH}}^{2}{}^{+}\to \mathrm{TiOH}+{\text{H}}^{+}$$

(4) $$\mathrm{TiOH}\to {\mathrm{TiO}}^{-}+\text{H}$$

Positive holes are the predominant oxidation species at low pH while ^·^OH are abundant in wastewater at high and neutral pH [17,18].

#### Effect of TiO_2_ concentration on photocatalytic degradation of COD using solar irradiation

**Figure 5:**
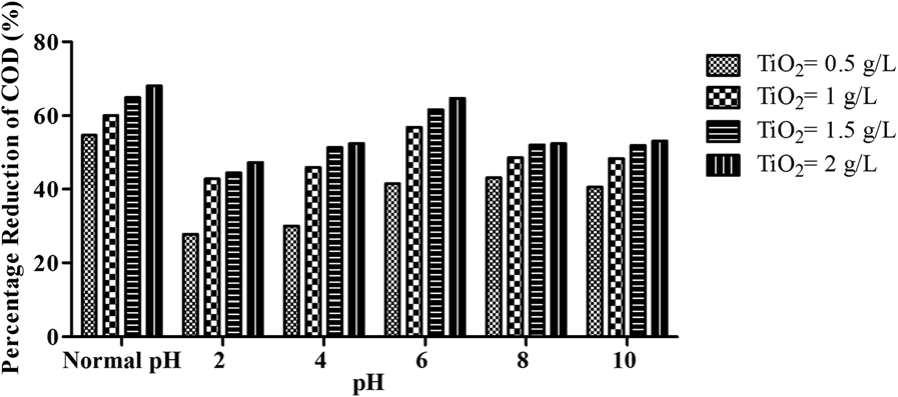
**Effect of pH on percentage reduction of COD using solar radiation. **Plot of COD reduction versus pH; initial concentration = >7500 mg/L; TiO_2_ = variable; average solar intensity = 80 W/m^2^; average temperature 32°C.

Generally, an increase in catalyst concentration results in a very rapid increase in degradation, which confirms to a heterogeneous regime [17]. The catalyst concentration was varied as 0.5, 1, 1.5 and 2 g/L to obtain an optimum concentration to be used for further experiments. pH value was also altered for all the catalyst doses. Experiments were performed at Normal, 2, 4, 6, 8 and 10 pH values for all the mentioned catalyst concentrations. The results are shown in Figure 5.

Increase in concentration of TiO_2_ increases the number of active sites on the photocatalyst surface, which in turn increases the number of OH° radicals. A reverse effect occurs when the TiO_2_ concentration increases to higher than the optimum value, the degradation rate declines due to the interference of the light by the suspension [19-21].

At the normal pH value of wastewater and a catalyst concentration of 0.5 g/L, 54% decrease in COD was observed in 240 minutes of reaction time period and no further reaction occurred. On increasing the concentration of catalyst to 1 g/L, 60% COD decrease was observed in 180 minutes of time. No further decrease was observed. Similar trends were observed on increasing the concentration of catalyst to 1.5 and 2 g/L, in which 64% and 68% COD decrease was observed in 180 minutes stopping the reaction thereafter. The rate constant values in Table 3 were found to increase upto TiO_2_ concentration of 1.5 g/L. No significant increase is observed for 2 g/L of TiO_2_. Possibly, high TiO_2_ concentration hinders the light penetration, thereby decreasing the reaction rate.

**Table 3:** **Variation of first order rate constant for COD reduction at different pH and TiO**_**2 **_**concentrations**

**Sample**	**TiO**_**2**_**Concentration (g/L)**	**First order rate constant (1/min)**					
		**Normal pH**	**pH = 2**	**pH = 4**	**pH = 6**	**pH = 8**	**pH = 10**
**COD**	**0.5**	0.0029	0.0012	0.0014	0.0019	0.0022	0.0018
	**1**	0.0035	0.0020	0.0023	0.0031	0.0025	0.0024
	**1.5**	0.0039	0.0021	0.0026	0.0036	0.0027	0.0027
	**2**	0.0040	0.0023	0.0027	0.0038	0.0027	0.0028
	**R**^**2**^	0.985	0.967	0.983	0.988	0.979	0.997

At pH = 2, COD decrease was very low. At 0.5 g/L of catalyst concentration, 25.5% of COD decrease was observed in 180 minutes. No significant reaction was observed after this time. At 1 g/L of catalyst concentration, 40% decrease was observed in 180 minutes with a slow increase upto 42.8% in 240 minutes. Similarly a maximum of 44.4% and 47.2% reduction was observed in 240 minutes in 1.5 and 2 g/L of catalyst concentration with 43% and 44.4% decrease in 180 minutes respectively. The rate constant values at pH = 2 (Table 3), indicate that an increase of rate upto 1 g/L of catalyst concentration. No further increase in rate was observed with increasing TiO_2_ dose, indicating that pH was the governing factor in determining the reaction rate.

On increasing the pH value to 4, overall 30% COD decrease was observed in 180 minutes and no further reaction was seen at 0.5 g/L of catalyst concentration. At 1 and 1.5 g/L of catalyst concentration, 46% and 51% COD decrease was observed in 240 minutes. At a catalyst concentration of 2 g/L, 51% decrease occurred in 180 minutes and no further significant reaction occurred. The rate constant values are also low as observed at pH = 2.

At pH value of 6, 41.5%, 57%, 61.6% and 64.6% of overall COD decrease was observed in 240 minutes at the catalyst concentration of 0.5, 1, 1.5 and 2 g/L respectively. No further decrease in COD was observed upto 300 minutes. The decrease was steady with respect to time. In a similar study, the maximum degradation was observed at pH = 6 [22]. The rate constant values increased significantly on increasing the TiO_2_ dose from 0.5 to 1 g/L. However, not much increase in rate was observed for 1.5 and 2 g/L of catalyst concentrations.

At pH = 8, reaction was slightly faster as compared to pH = 6. A COD decrease of 41%, 47%, 51% and 52% was observed in 180 minutes for 0.5, 1, 1.5 and 2 g/L catalyst concentrations respectively. No significant decrease was observed on further solar exposure upto 300 minutes. The rate constant values indicate no significant improvement with the TiO_2_ dose. The overall rates were less than those observed at pH = 6 and at normal pH values.

At pH 10 and 0.5 g/L of catalyst concentration, COD decrease of 37.5% in 240 minutes and 40.6% in 300 minutes was observed. For 1, 1.5 and 2 g/L, 48%, 52% and 53% COD decrease was observed respectively in 240 minutes, indicating a slow and almost similar reaction rate at pH = 8 for these catalyst concentrations. First order rate constant values at pH = 10 (Table 3) were nearly similar to those obtained for pH = 8.

An overall assessment of this study shows that pH plays even a more significant role than catalyst dose or other factors. Maximum and fastest reaction was observed at Normal pH values (pH = 6.8) even at 0.5 g/L of catalyst concentration. A similar trend of maximum COD decrease at normal pH was observed in absence of TiO_2._ Acidic condition was less favourable than normal and alkaline pH due to amphoteric behaviour above and below point of zero charge (pzc) [23]. The pzc value was found at pH = 6.25. The TiO_2_ surface is positively charged in acidic media (pH < 6.25), whereas it is negatively charged under alkaline conditions (pH > 6.25).

The reaction rates are dominated by pH alterations. Nearly similar trends of decrease were observed at concentration. 0.5, 1, 1.5 and 2 g/L for all pH values. No doubling effect of catalyst was observed which prompted us to select 1 g/L as an optimum concentration for further experimental runs.

### Effect of H_2_O_2_ concentration

#### Effect of H_2_O_2_ concentration on dark absorption of COD

**Figure 6:**
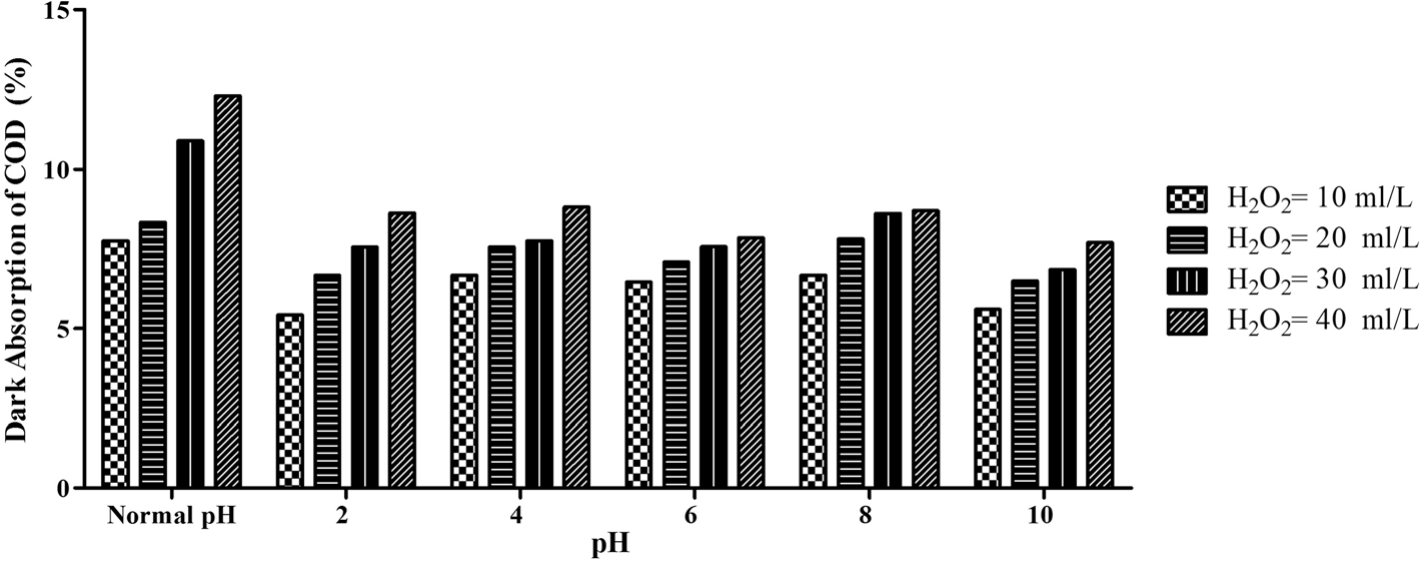
**Dark adsorption of organics at different pH values; initial COD concentration = >7500 mg/L; H**_**2**_**O**_**2**_ **= variable; TiO**_**2**_ **= 1 g/L; average temperature = 23°C.**

The beneficial role of H_2_O_2_ in photo reduction was found to be remarkable; however there are other, more subtle effects. For example, the amount of COD initially absorbed on TiO_2_ (in the dark) was affected as the H_2_O_2_ concentration was increased. This trend is given in Figure 6. Dark adsorption was found to increase with increasing H_2_O_2_ concentration.

The low adsorption obtained might be due to the adverse effect of excess H_2_O_2_ on the reaction as described, that H_2_O_2_ consumes generated ^·^OH if the optimal dosage is not computed and employed [24,25]. At Normal pH, 7.7% and 8.3% dark absorption was observed at the H_2_O_2_ concentrations of 10 and 20 mL/L respectively. At 30 mL/L and 40 mL/L of H_2_O_2_ concentration, 10.8% and 12.3% adsorption of organics was observed.

At pH = 2, 5.4%, 6.6% and 7.5% dark absorption at the H_2_O_2_ concentrations of 10 mL/L, 20 mL/L and 30 mL/L and 40 mL/L of H_2_O_2_ concentration the reaction was faster and 8.6% adsorption was observed.

At pH = 4, a 6.6% decrease at H_2_O_2_ concentration of 10 mL/L was observed. On increasing the concentration to 20 mL/L and 30 mL/L, dark absorption was 7.5% and 7.7% respectively in 240 minutes. At 40 mL/L of H_2_O_2_ concentration, 8.8% adsorption was seen in 240 minutes which did not increase further even after 240 minutes.

At pH = 6, a dark absorption of 6.4%, 7.08%,7.5% and 7.8% was observed in 240 minutes for 10, 20, 30 mL/L and 40 mL/L of H_2_O_2_ concentration respectively. No further reaction was observed after 240 minutes.

At pH = 8, a maximum of 6.6% and 7.8% absorption of COD decrease were observed in 240 minutes at 10 and 20 mL/L of H_2_O_2_ concentrations respectively. At 30 mL/L and 40 mL/L of H_2_O_2_ concentration, a maximum of 8.6% and 8.7% decrease was observed respectively in 180 minutes.

At pH value of 10, adsorption of 5.5%, 6.4%, 6.8% and 7.6% was observed in 240 minutes for 10, 20, 30 and 40 mL/L H_2_O_2_ concentrations respectively.

The trends of dark adsorption were similar to that of photocatalytic reaction.

#### Effect of H_2_O_2_ concentration on photocatalytic reduction of COD using solar irradiation

**Figure 7:**
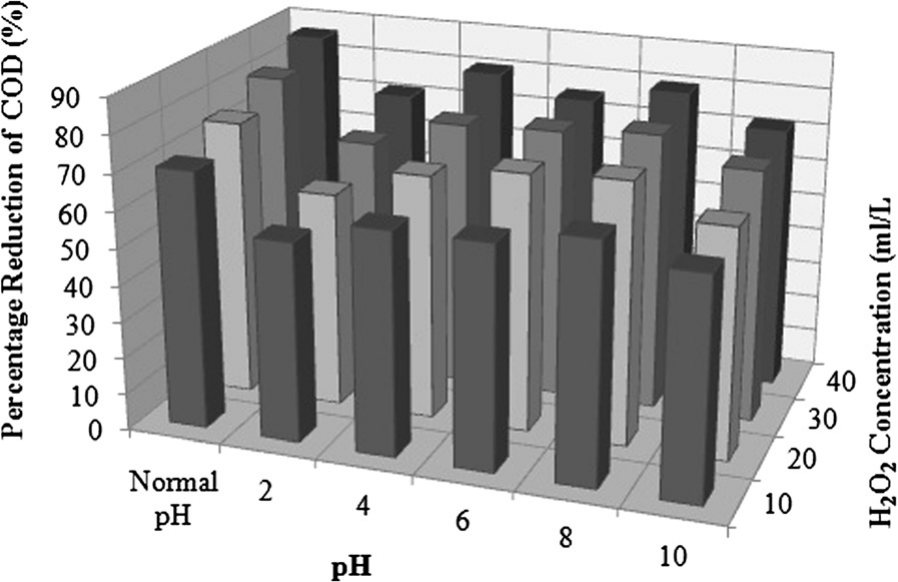
**Effect of pH on percentage reduction of COD in presence of H**_**2**_**O**_**2 **_**using solar radiation. **Plot of COD reduction versus pH; Initial concentration = >7500 mg/L; H_2_O_2_ = variable; TiO_2_ = 1 g/L; average solar intensity = 80 W/m^2^; average temperature = 23°C.

H_2_O_2_ was added as oxidizing agent to enhance the reaction rate. Concentration of H_2_O_2_ was varied to 10, 20, 30 and 40 mL/L. The results are given in Figure 7.

At normal pH and 10 mL/L of H_2_O_2_, 68% of COD decrease was observed in 240 minutes which increased to 70.5% in 300 minutes. On increasing the concentration of H_2_O_2_ to 20 mL/L, 74.4% COD decrease was observed in 240 minutes. No significant reduction was observed after that. Further on increasing the concentration to 30 mL/L, the reaction became faster. A 69.4% COD decrease was observed in 180 minutes of solar exposure, which further increased to 81% in 240 minutes.

On adding 40 mL/L of H_2_O_2_, a maximum decrease of 86.3% was observed in 180 minutes of time period. However, the reaction halted after that indicating a possible consumption of H_2_O_2_. The rate constant values in presence of H_2_O_2_ (Table 4) were highest for the normal pH values among all the experiments performed. The values increased steadily with an increasing concentration of H_2_O_2_, with a maximum of the order of 0.0076 1/min.

**Table 4:** **Variation of first order rate constant for COD reduction at different pH and H**_**2**_**O**_**2 **_**concentrations, TiO**_**2**_ **= 1 g/L)**

**Sample**	**H**_**2**_**O**_**2**_**Concentration (mL/L)**	**First order rate constant (1/min)**					
		**Normal pH**	**pH = 2**	**pH = 4**	**pH = 6**	**pH = 8**	**pH = 10**
**COD**	**0**	0.00347	0.00203	0.00227	0.00313	0.00246	0.00245
	**10**	0.00439	0.00299	0.00376	0.00365	0.00415	0.00344
	**20**	0.00535	0.00329	0.00431	0.00464	0.00486	0.00367
	**30**	0.00641	0.00388	0.00505	0.00512	0.00548	0.00441
	**40**	0.00755	0.00467	0.00619	0.00507	0.00619	0.00472
	**R**^**2**^	0.93329	0.79809	0.85309	0.98241	0.93757	0.80822

At pH = 2, 54.7%, 58.9% and 65% COD decrease was observed at the H_2_O_2_ concentrations of 10, 20 and 30 mL/L. At 40 mL/L of H_2_O_2_ concentration the reaction was faster, 71.5% COD decrease was observed in 180 minutes of time period. Rate constant value of 0.0047 1/min was observed for 40 mL/L of H_2_O_2_. In spite of acidic pH values, which showed overall slow reaction rates for all the experiments performed, a doubling of rate was observed in presence of H_2_O_2_ even for this pH value.

At pH = 4, a 57.8% decrease in 180 minutes and 61% in 240 minutes at H_2_O_2_ concentration of 10 mL/L was observed. On increasing the concentration to 20 mL/L, COD decrease was 60.6% in 180 mins which further increased to 67% in 240 minutes. On increasing the concentration to 30 mL/L, 67.7% decrease was observed in 180 minutes which increased to 73% in 240 minutes. At 40 mL/L of H_2_O_2_ concentration, 71.7% COD decrease occurred in 180 minutes which increased to 80.4% in 240 minutes.

At pH = 6, a COD decrease of 61%, 70.5% and 74% was observed in 240 minutes for 10, 20 and 30 mL/L of H_2_O_2_ concentration respectively. At 40 mL/L of H_2_O_2_, the reaction was fastest with 75% of COD decrease in 180 minutes.

At pH = 8, 57.8% COD decrease was observed in 180 minutes which further increased to 64.2% in 240 minutes. On doubling the H_2_O_2_ concentration, 59% decrease occurred in 180 minutes which further increased to 71.5% in 240 minutes. On tripling the H_2_O_2_ concentration a maximum COD decrease of 69.5% occurred in 180 minutes which was merely increased to 75.7% in 240 minutes. At 40 mL/L of H_2_O_2_, 76.8% COD decrease occurred in 180 minutes which increased just to 80% in 240 minutes.

At pH = 10, a maximum of 58.9% and 62.7% COD decrease was observed in 240 minutes at 10 and 20 mL/L of H_2_O_2_ concentrations respectively. At 30 mL/L and 40 mL/L of H_2_O_2_ concentration, a maximum of 69% and 71.5% decrease was observed respectively in 180 minutes.

Rate constant values at pH = 4, 6 and 8 were quite similar being maximum 0.0062 1/min at 40 mL/L of H_2_O_2_. At pH 10 and pH = 4 the rate of reaction was slow and nearly similar rate constant values were obtained for both the pH values. Increase in the degradation efficiency under the alkaline conditions could be attributed to the increase in hydroxyl ions, which induce more hydroxyl radical formation. In acidic condition, the per-hydroxyl radical can form hydrogen peroxide, which in turn gives rise to the hydroxyl radical [26], making the reaction slower.

Presence of H_2_O_2_ significantly enhanced the reaction rate and percentage decrease of organic content was significantly higher as compared to reactions without H_2_O_2_.

### Effect of solar intensity

Although the maximum intensity of solar radiations was available in the initial 2-4 hours, the maximum reduction was obtained in first two hours of solar exposure in all the experimental run. It can be presumed that the reaction was performed efficiently at the solar irradiation between 600-865 W/m^2^ (Tables 5 and 6).

**Table 5:** **Solar irradiation (average of 5 h), pH and maximum temperature attained for experimental runs at different pH values and TiO**_**2 **_**dose (corresponding of Figures **3**and **5**)**

**Concentration of TiO**_**2**_**(g/L)**	**pH**	**Initial pH value**	**Final pH value**	**Average solar irradiation (W/m**^**2**^**)**	**Maximum temperature attained (°C)**
**Without TiO**_**2**_	**Normal pH**	6.8	7.32	836	49
	**2**	2	2.05	788	44
	**4**	4	4.13	788	46
	**6**	6	6.31	836	50
	**8**	8	7.15	704	34
	**10**	10	9.02	704	38
**With TiO**_**2**_**(0.5 g/L)**	**Normal pH**	5.5	6.9	820	47
	**2**	2	2.1	850	48
	**4**	4	4.05	760	42
	**6**	6	6.1	743	37
	**8**	8	5.72	697	36
	**10**	10	6.45	600	33
**TiO**_**2**_**(1 g/L)**	**Normal pH**	5.87	6.49	664	33
	**2**	2	2.06	850	48
	**4**	4	4.08	760	42
	**6**	6	6.04	743	37
	**8**	8	6.07	697	36
	**10**	10	5.95	600	33
**TiO**_**2**_**(1.5 g/L)**	**Normal pH**	7.05	6.8	816	38
	**2**	2	2.09	837	32
	**4**	4	4.06	760	42
	**6**	6	6.06	743	37
	**8**	8	5.85	747	39
	**10**	10	6.33	627	33
**TiO**_**2**_**(2 g/L)**	**Normal pH**	7.05	6.71	664	36
	**2**	2	2.13	837	46
	**4**	4	4.06	760	43
	**6**	6	6.07	743	39
	**8**	8	6.92	747	40
	**10**	10	6.22	627	36

**Table 6:** **Average solar irradiation, temperature attained for experiment runs at fixed TiO**_**2 **_**(1 g/L) different hydrogen peroxide (H**_**2**_**O**_**2**_**) concentration and pH in 5 hours (corresponding to Figure **7**)**

**Different pH**	**H**_**2**_**O**_**2**_**Concentration (mL/L)**	**Initial pH value**	**Final pH value**	**Average soar irradiation (W/m**^**2**^**)**	**Average temperature attained (C°)**
**Normal pH**	**10**	7.05	6.86	864	29
	**20**	7.05	6.49	864	29
	**30**	7.05	6.80	864	26
	**40**	7.05	6.71	864	26
**pH = 2**	**10**	2	1.98	864	29
	**20**	2	1.95	864	30
	**30**	2	2.18	864	26
	**40**	2	2.10	864	26
**pH = 4**	**10**	4	3.89	212	37
	**20**	4	4.00	212	37
	**30**	4	2.62	212	36
	**40**	4	2.56	212	37
**pH = 6**	**10**	6	5.13	786	36
	**20**	6	4.88	786	38
	**30**	6	5.00	786	40
	**40**	6	4.98	786	39
**pH = 8**	**10**	8	5.83	801	33
	**20**	8	5.39	801	34
	**30**	8	5.24	801	32
	**40**	8	5.14	801	35
**pH = 10**	**10**	10	3.62	811	39
	**20**	10	3.35	811	39
	**30**	10	3.12	811	41
	**40**	10	2.99	811	38

Some replicate experimental run performed on a cloudy day, (solar irradiation 200-250 W/m^2^), showed low significant COD reduction in 5 hours. It is reported [27] that above a certain value (estimated to be 25 W/m^2^ of UV in laboratory experiments); the reaction rate becomes proportional to the square root of UV intensity.

### Kinetic modeling of the photocatalytic process

It was tried to verify [28] if the reaction was in accordance with the LH mechanism. According to the L-H model confirming the heterogeneous catalytic character of the system with the rate ‘r’ varying proportionally with the surface coverage θ as [29,30]:

(5) $$\text{r}=\mathrm{k\theta}=\frac{\text{k}\left(\mathrm{KG}\right)}{1+\mathrm{KC}}$$

Where ‘r’ is the rate of reaction, ‘K’ is the adsorption constant, ‘k’ is the rate constant and ‘C’ is the concentration of the species. The degradation rate constant (k) is determined and evaluated at different H_2_O_2_ concentrations to find out the values at which maximum efficiency (k_max_) is obtained. In all the following experiments is described assuming a pseudo-first order reaction. Then, an approximation of L-H expression can be used [31]:

(6) $$\frac{-\text{d}\left(\text{C}\right)}{\mathrm{dt}}=-\text{k}\left(\text{C}\right)$$

Where, k is the pseudo-first order reaction rate constant. Integration of equation 7 leads to:

(7) $$\frac{\ln\left(\text{c}\right)}{\mathrm{CO}}=-\mathrm{kt}$$

From where the slope of the plot ln (C)/Co vs. t (time of irradiation) renders the reaction rate constant (k).

Since the rate of reduction was found to varied with the initial concentration of H_2_O_2_, the initial rate determined for COD reduction are compared with the increasing concentration. The L-H plots the values of slope (R^2^) and equation for the trend line with respect to increase in H_2_O_2_ concentrations. A straight line was obtained on plotting the 1/rate versus 1/concentration of TiO_2_ and H_2_O_2_ separately. It can be observed that the L-H mechanism was followed with respect to increasing H_2_O_2_ concentrations in the wastewater shown in (Tables 3 and 4) with high correlation coefficients.

## Conclusion

Solar detoxification process was effective in treating a wastewater with a COD level of the order of more than 7500 mg/L, which is otherwise a difficult waste to treat. It can therefore be used as a treatment step in the high organic wastewater treatment during the primary stage also as it effectively reduces the COD content by 86%. The rate of degradation is faster with concentrated solar radiation. pH was found be a dominant factor affecting reaction rate even in presence of additional oxidant. Maximum reduction of organic content was observed at the normal pH value of the wastewater (pH = 6.8) for all the experimental runs. Therefore there would be no requirement of any pH alteration for wastewater treatment by this technique. Thus, solar detoxification of high COD wastewater is a unique treatment process utilizing renewable solar energy and treating the wastewater with minimum chemical input.

## Competing interests

The authors are thankful to the Ministry of New and Renewable Energy (MNRE), TERI University, New Delhi, India, for providing the financial support for the research.

## Authors’ contributions

The overall implementation of this study including experiments, data analysis, and manuscript preparation were the results of joint efforts by individuals who are listed as co-authors of this paper. All authors have made extensive contribution into the review and finalization of this manuscript. All authors read and approved the final manuscript.
